# Coagulation markers as independent predictors of colorectal cancer aggressiveness

**DOI:** 10.1186/s12876-025-04249-4

**Published:** 2025-09-02

**Authors:** Hanaa Ali EL-Sayed, Doaa H. Sakr, Mostafa abdelhakiem, Mohamed Awad Ebrahim, Maha Othman, Hanan Azzam

**Affiliations:** 1 Clinical Pathology Department, Faculty of Medicine, Mansoura University, Mansoura, Egypt; 2 Oncology Department, Faculty of Medicine, Mansoura University, Mansoura, Egypt; 3 Department of Biomedical and Molecular Sciences, Queen’s University, Kingston, ON Canada

**Keywords:** Colorectal cancer, D-dimer, Fibrinogen, Hemostasis, Prothrombin time

## Abstract

**Background:**

Colorectal cancer (CRC) is frequently associated with thrombosis with thrombotic events, such as deep vein thrombosis or pulmonary embolism, often correlate with poor clinical outcomes. Coagulation markers have been suggested as potential prognostic indicators for CRC severity. However, the relationship with clinicopathological characteristics in CRC remains unclear.

**Purpose:**

This study aims to examine the relationship between routine coagulation markers and clinicopathological characteristics in CRC patients.

**Patients and methods:**

A retrospective analysis was conducted on 100 patients with confirmed diagnosis of CRC, classified according to the 2018 edition of the American Joint Committee on Cancer Tumor/Node/Metastasis staging system for malignant tumors. Clinicopathological characteristics and routine coagulation tests including prothrombin time, and international normalized ratio, activated partial thromboplastin time, prothrombin activity, thrombin time, fibrinogen, d-dimer, platelet count, were evaluated. Spearman correlation was used to assess correlations with clinicopathological characteristics. Additionally, univariate and multivariate ordinal regression analysis were conducted to detect the independent predictors for CRC aggressiveness.

**Results:**

Our data documents several associations between coagulation markers and cancer progression markers. Specifically, positive correlations were identified between fibrinogen and d-dimer levels and each of the following: carcinoembryonic antigen, carbohydrate antigen, tumor stage, node involvement, and metastasis. Regression analysis showed, d-dimer (OR = 1.102, *p* < 0.001) and fibrinogen (OR = 1.002, *p* < 0.001) are independent predictors of high-risk CRC cases.

**Conclusion:**

Fibrinogen and d-dimer may serve as independent predictive biomarkers for CRC aggression. Their clinical utility could support personalized treatment plans for CRC patients.

**Supplementary Information:**

The online version contains supplementary material available at 10.1186/s12876-025-04249-4.

## Introduction

Colorectal cancer (CRC), encompassing both colon and or rectal cancer, is a major health concern ranking as the third most commonly diagnosed and second most deadly cancer globally [1]. In 2020, CRC accounted for 9.4% of all cancer-related deaths [2]. With an increasing incidence particularly among elderly population, global CRC cases are projected to more than double by 2035, highest surge in less developed countries [3].

Early detection of CRC relies on various diagnostic techniques, including invasive and non-invasive methods. While molecular and genetic characterizations of tumors offer advanced diagnostic potential, they are still used alongside conventional methods, often resulting in suboptimal detection and poor long-term survival rates. Biomarkers such as miRNA, play a crucial role in improving diagnostic accuracy [4].

Abnormal hemostasis function has been strongly linked to cancer development [5]. Tumors can activate hameostasis-related processes in surrounding tissues, promoting metastasis and tumor growth. The level of activation has been linked to metastasis and tumor cell growth [6, 7]. Dysregulated coagulation contributes to malignant proliferation, enhances tumor adhesion to blood vessels, stimulates angiogenesis, and aids immune evasion [8]. The hypercoagulable state in cancer patients reflects tumor biology, making coagulation parameters potential markers for tumor risk classification [9].Coagulation markers have been significantly associated with the clinicopathological features of various cancers, including lung, breast, and stomach cancer [10, 11]. However, limited studies have explored their role in CRC. This study aims to assess key coagulation markers—fibrinogen, d-dimer, activated partial thromboplastin time, prothrombin time, prothrombin activity, thrombin time, platelet count, and international normalized ratio—to evaluate their potential in determining CRC aggressiveness. By examining their correlation with clinicopathological features, we aim to highlight their clinical utility in CRC prognosis and risk assessment.

## Patients and methods

### Patients

Between January 2023 and October 2024, 219 cases were diagnosed with colorectal cancer (CRC) at the Oncology Centre of Mansoura University (OCMU). Strict inclusion requirements were set in order to guarantee the validity and reliability of the results. The requirements for inclusion were as follows: Cases who (1) had complete case data at diagnosis and treatment, (2) had a pathological examination and were diagnosed with CRC, (3) did not have any other primary malignancies diagnosed or treated concurrently or previously, (4) did not receive chemotherapy or radiation therapy prior to treatment, (5) did not have autoimmune diseases, hepatic diseases, blood & vascular disorders that could have an impact on coagulation parameters, (6) cases without a history of significant trauma or major surgery, and (7) cases who had not taken anticoagulant medication one week before to the visit. In order to ensure that the study cases shared a common medical history and that their coagulation parameters were undisturbed by outside influences, these criteria were carefully selected. Applying these standards would give future research analyses a more precise and trustworthy foundation. One hundred CRC cases were chosen for this study after our inclusion criteria were strictly followed. A retrospective review of preoperative imaging and laboratory results, postoperative pathological findings, and baseline characteristics was conducted.

### Data collection and ethical approval

Clinicopathological data from 100 CRC cases were retrospectively collected, including preoperative laboratory tests, imaging results, and postoperative pathology findings. Baseline factors such as age, smoking history, diabetes, and hypertension were also recorded (Table 1). Colorectal biopsies were assessed by the pathology unit, with relevant clinical data retrieved from medical records. Histological grading followed the WHO system based on gland formation percentage [12], while staging adhered to the 2018 AJCC TNM classification [13]. The study was approved by Mansoura University's Faculty of Medicine Institutional Review Board (R.24.10.2856.R1.R1) and conducted per ethical guidelines. Informed consent was obtained from all participants before inclusion.

**Table 1:** Clinicopathological characteristics and laboratory data of all CRC cases

	All patients N = 100
Male, n(%)	45(45%)
Female, n(%)	55(55%)
Age (years), mean ± SD (range)	53.38 ± 14.64 (18–77)
*Site, n(%)*	
Colon	62 (62%)
Rectum	38 (38%)
*Smoking, n(%)*	
Positive	27 (27%)
Negative	73 (73%)
*Hypertension, n(%)*	
Positive	36 (36%)
Negative	64 (64%)
*Diabetes, n(%)*	
Positive	18 (18%)
Negative	82 (82%)
*Tumor stage, n(%)*	
T1	3 (3%)
T2	8 (8%)
T3	61 (61%)
T4	28 (28%)
*Node stage, n(%)*	
N0	28 (28%)
N1	37 (37%)
N2	34 (34%)
N3	1 (1%)
*Metastasis stage, n(%)*	
M0	86 (86%)
M1	14 (14%)
*Stage, n(%)*	
I	3 (3%)
II	27 (27%)
III	56 (56%)
IV	14 (14%)
*Histological Grade, n(%)*	
G1	2 (2%)
G2	71 (71%)
G3	16 (16%)
G4	11 (11%)
CA19-9 (U/ml), median (range)	213 (13–3400)
CEA (ng/ml), median (range)	213 (13–1800)
Platelet count (× 10^9^/L), median (range)	286.5 (53–937)
Prothrombin time (s), median (range)	12 (12–15.5)
Prothrombin activity (%), median (range)	100 (65.9–100)
International normalized ratio, median (range)	1 (1–1.4)
Activated partial thromboplastin time (s), median (range)	35.5 (33–40)
Thrombin time (s), median (range)	20 (18–20)
Fibrinogen (mg/dl), median (range)	382 (210–567)
D-dimer (mg/l), median (range)	4.3 (0.6–12)

Categorical data are expressed as count (%); parametric data are expressed as mean ± SD (minimum–maximum); non parametric data are expressed as median (minimum–maximum)

### Coagulation and other tests

Three days before surgery, all patients had blood tests. Venous blood samples were collected in citrated tubes for the coagulation and fibrinolysis markers. Centrifugation at room temperature for 15–20 min (speed: 3500 × g) was used to obtain platelet-poor plasma. Prothrombin time and activated partial thromboplastin time testing were performed right away. Without delay, aliquots of plasma were moved to plastic tubes and frozen at − 80 °C until fibrinogen and d-dimer analysis. A Sysmex CS-2500 coagulation analyzer (Sysmex) was used to identify the traditional coagulation biomarkers prothrombin time, prothrombin activity, thrombin time, fibrinogen, d-dimer, activated partial thromboplastin time, and international normalized ratio using the appropriate assay kits. Using the appropriate assay kits, the Roche Cobas e411 (Roche, Switzerland) was used to measure the tumor biomarkers carcinoembryonic antigen (CEA) and carbohydrate antigen (CA) 19–9. A Sysmex hematology analyzer with the appropriate kits was used to measure the complete blood cell counts, which were ascertained using an automatic cell counter with EDTA blood.

### Statistical analysis

The Shapiro–Wilk test assessed the normality of quantitative variables. For normally distributed variables, independent-sample T-tests or one-way ANOVA were applied, with results presented as mean ± standard deviation (SD). Non-normally distributed variables were analyzed using the Mann–Whitney U or Kruskal–Wallis H tests, with results shown as medians (range). Categorical variables were compared using the chi-square test and expressed as frequencies and percentages.

CRC cases were classified according to the 2018 AJCC/TNM staging system [13], and clinicopathological and coagulation differences among stages were evaluated. Fibrinogen levels were divided into tertiles: C1 (< 345 mg/dL, n = 37), C2 (346–456 mg/dL, n = 30), and C3 (> 457 mg/dL, n = 33), and their associations with clinicopathological and coagulation features were analyzed. Similarly, d-dimer levels were stratified into G1 (< 3.5 g/L, n = 35), G2 (3.6–7.3 g/L, n = 33), and G3 (> 7.4 g/L, n = 32), and their correlations were examined.

Univariate ordinal regression assessed the relationship between coagulation markers and CRC severity, while multivariate regression identified independent predictors of advanced-stage CRC. Statistical analyses were conducted using SPSS (version 26.0, IBM Corp.), with a significance threshold of *P* < 0.05 for all two-sided tests.

### Data availability statement

The datasets used and/or analysed during the current study available from the corresponding author on reasonable request.

## Results

The study included 100 cases with a mean age of 53.38 years (range 18–77). Hypertension was present in 75%, diabetes in 18%, and smoking in 27%. Tumor staging revealed 3% in T1, 8% in T2, 61% in T3, and 28% in T4, with 14% having distant metastases and 72% showing lymph node involvement. Tumor grading categorized 2% as grade 1, 71% as grade 2, 16% as grade 3, and 11% as grade 4. Laboratory findings showed median CA19-9 and CEA levels 213 U/mL (13–3400) and 213 ng/mL (13–1800), respectively. Median of platelet count was 286.5 × 10^9^/L (53–937). According to coagulation markers, the median of prothrombin time was (12 s), international normalized ratio was (1.0), thrombin time was (20 s). d-dimer was 4.3 mg/L (0.6–12), and fibrinogen was 382 mg/dL (210–567) (Table 1).

TNM staging categorized patients as early-stage (I & II, 30%), node metastasis (III, 56%), and distant metastasis (IV, 14%). Significant differences (*p* < 0.05) were observed between these groups in smoking, hypertension, diabetes, CA19-9 & CEA levels, histological grade, platelet count, activated partial thromboplastin time, fibrinogen, and d-dimer. Notably, d-dimer and fibrinogen were significantly higher in node and distant metastasis groups than in early-stage cases. No statistical significant differences were found between these groups (*P* > 0.05) according to gender, age, site, prothrombin time and INR (Supplemental Table 1 & Supplemental Fig. 1).

**Fig. 1:**
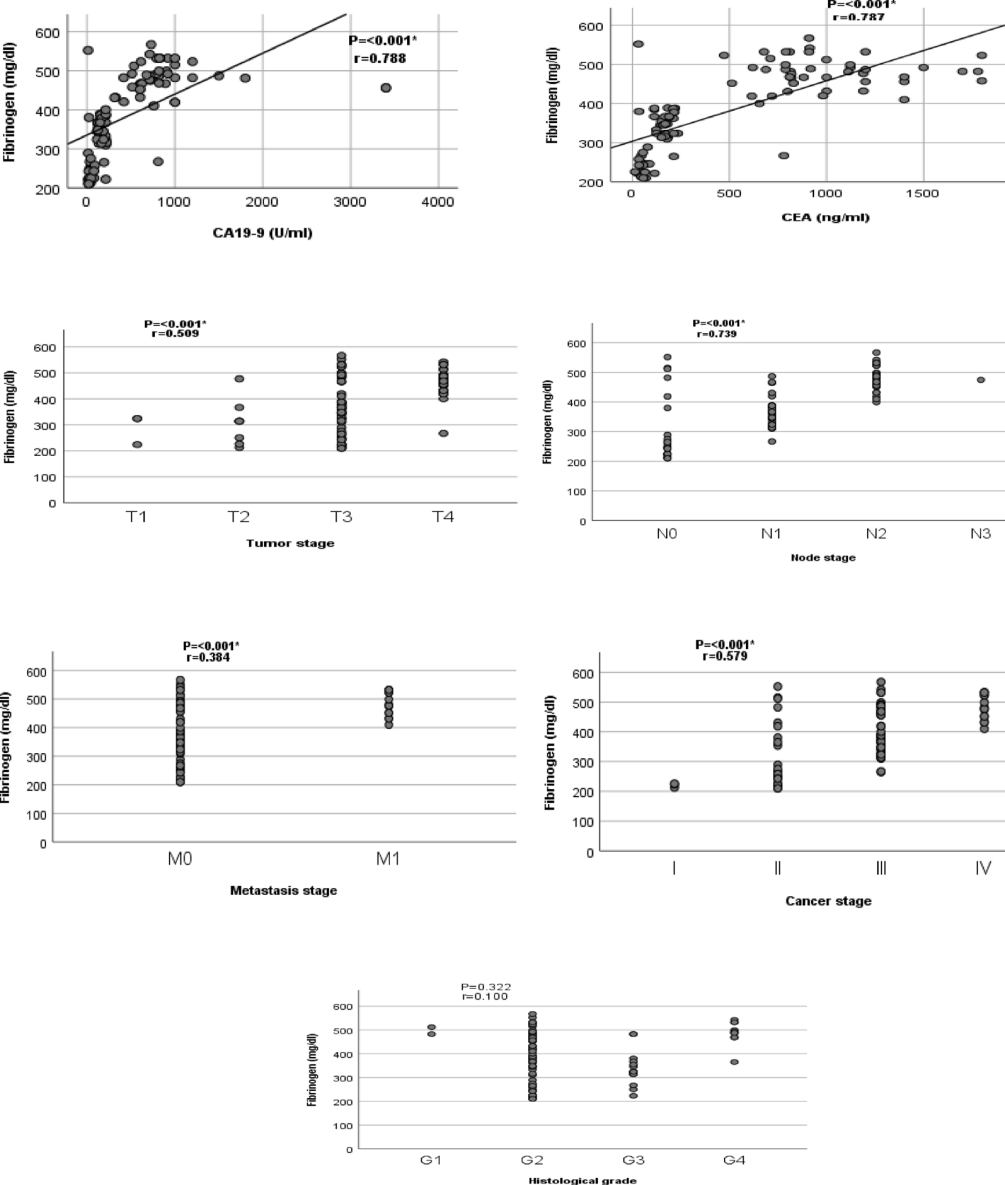
Correlation between fibrinogen and clinicopathological traits of CRC cases

**Fig. 2:**
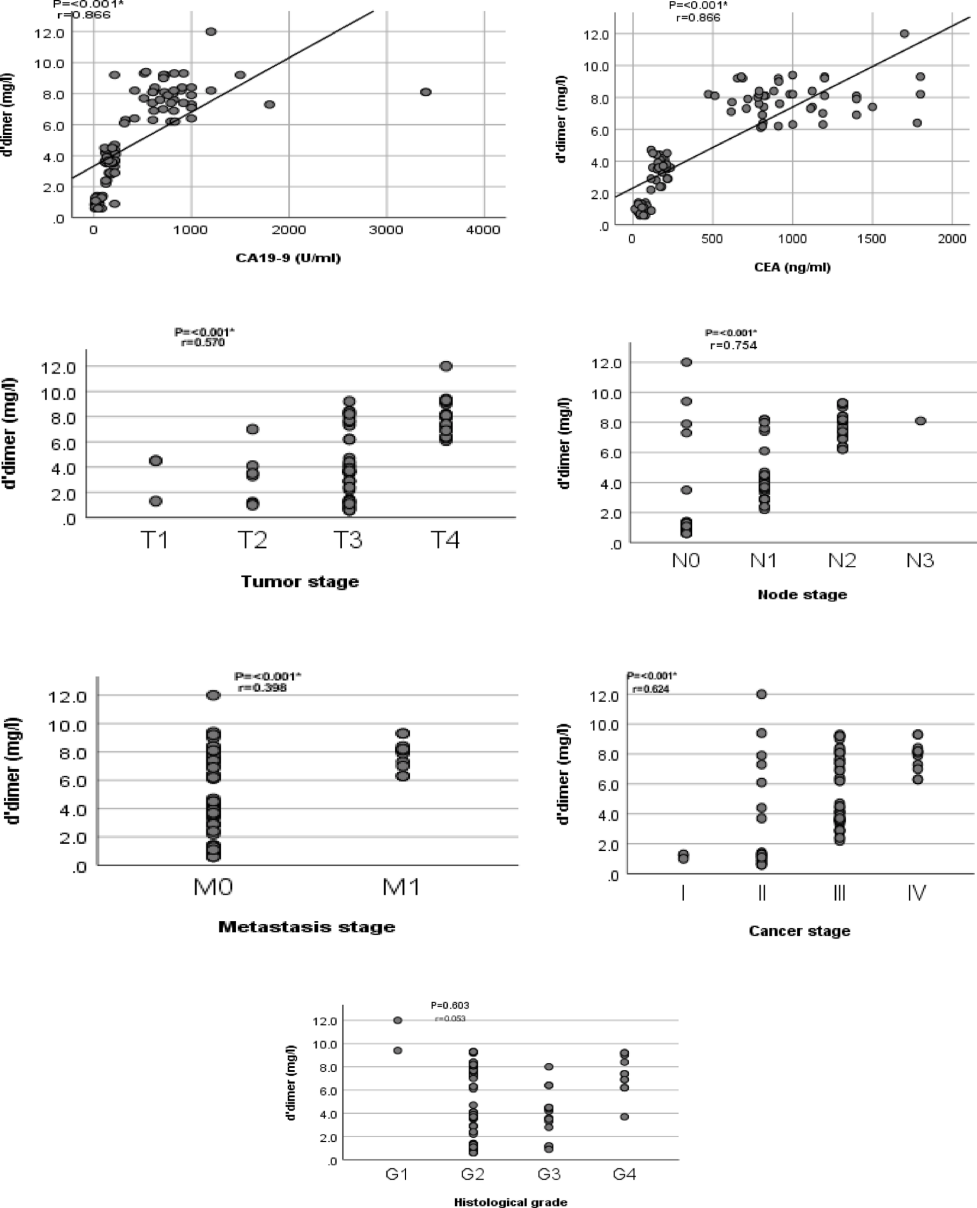
Correlation between d-dimer and clinicopathological traits of CRC cases

Spearman’s correlation analysis indicated strong positive associations between plasma fibrinogen and CA19-9 (r = 0.788, *p* < 0.001), CEA (r = 0.787, *p* < 0.001), tumor stage (r = 0.509, *p* < 0.001), node stage (r = 0.739, *p* < 0.001), and metastasis stage (r = 0.384, *p* < 0.001), but not histological grade (r = 0.100, *p* = 0.322) (Fig. 1). Similarly, d-dimer levels correlated significantly with CA19-9 (r = 0.866, *p* < 0.001), CEA (r = 0.866, *p* < 0.001), tumor stage (r = 0.570, *p* < 0.001), and metastasis stage (r = 0.398, *p* < 0.001), but not histological grade (r = 0.053, *p* = 0.603) (Fig. 2).

Tertile-based grouping of fibrinogen and d-dimer levels revealed significant associations with CA19-9, CEA, histological grade, hypertension, tumor stage, lymph node metastasis, and distant metastasis (*p* < 0.05). The increase in fibrinogen and d-dimer (> 457 mg/d & > 7.4 g/l, respectively) showed statistical significant increase in clinicopathological features of CRC as regard tumor markers, histological grade and TNM staging (*p* < 0.05) (Tables 2 and 3 & Supplemental Figs. 2 and 3).

**Table 2:** Data of CRC cases categorized by tertiles of plasma fibrinogen levels

		Fibrinogen			*p*	Pairwise comparison
		C1 (< 345 mg/dl, n = 37)	C2 (346–456 mg/dl, n = 30)	C3 (> 457 mg/dl, n = 33)		
Male		10 (27%)	18 (60%)	17 (51.5%)	0.055	
Female		27 (73%)	12 (40%)	16 (48.5%)		
Age (years)		60 (36–74)	51 (18–73)	52 (27–77)	0.742	
Site	Colon	21 (56.8%)	21 (70%)	20 (60.6%)	0.529	
	Rectum	16 (43.2%)	9 (30%)	13 (39.4%)		
Smoking	Positive	9 (24.3%)	9 (30%)	9 (27.3%)	0.873	
	Negative	28 (75.7%)	21 (70%)	24 (72.7%)		
Hypertension	Positive	21 (56.8%)	9 (30%)	6 (18.2%)	0.003*	*P1* = 0.047* *P2* = 0.001* *P* = *0.376*
	Negative	16 (43.2%)	21 (70%)	27 (81.8%)		
Diabetes	Positive	5 (13.5%)	9 (30%)	4 (12.1%)	0.122	
	Negative	32 (86.5%)	21 (70%)	29 (87.9%)		
Tumor stage	T1	3 (8.1%)	0 (0%)	0 (0%)	< 0.001*	*P1* = 0.002* *P2* = < 0.001* *P3* = *0.342*
	T2	6 (16.2%)	1 (3.3%)	1 (3%)		
	T3	27 (73%)	19 (63.3%)	15 (45.5%)		
	T4	1 (2.7%)	10 (33.3%)	17 (51.5%)		
Node stage	N0	22 (59.5%)	2 (6.7%)	4 (12.1%)	< 0.001*	*P1* = < 0.001* *P2* = < 0.001* *P3* = < 0.001*
	N1	15 (40.5%)	19 (63.3%)	3 (9.1%)		
	N2	0 (0%)	9 (30%)	25 (75.8%)		
	N3	0 (0%)	0 (0%)	1 (3%)		
Metastasis stage	M0	37 (100%)	25 (83.3%)	24 (72.7%)	0.004*	*P1* = 0.010* *P2* = 0.001* *P3* = *0.373*
	M1	0 (0%)	5 (16.7%)	9 (27.3%)		
Stage	I	3 (8.1%)	0 (0%)	0 (0%)	< 0.001*	*P1* = 0.002* *P2* = < 0.001* *P3* = *0.573*
	II	18 (48.6%)	5 (16.7%)	4 (12.1%)		
	III	16 (43.2%)	20 (66.7%)	20 (60.6%)		
	IV	0 (0%)	5 (16.7%)	9 (27.3%)		
Histological Grade	G1	0 (0%)	0 (0%)	2 (6.1%)	< 0.001*	*P1* = *0.087* *P2* = < 0.001* *P3* = 0.015*
	G2	26 (70.3%)	26 (86.7%)	19 (57.6%)		
	G3	11 (29.7%)	3 (10%)	2 (6.1%)		
	G4	0 (0%)	1 (3.3%)	10 (30.3%)		
CA19-9 (U/ml)		56 (13–214)	213 (112–1800)	776 (213–3400)	< 0.001*	*P1* = 0.002* *P2* = < 0.001* *P3* = 0.001*
CEA (ng/ml)		65 (13–114)	210 (112–234)	912 (472–1800)	< 0.001*	*P1* = < 0.001* *P2* = < 0.001* *P3* = 0.006*
Platelet count (× 10^9^/L)		312 (127–937)	286 (53–747)	283 (153–619)	0.233	
Prothrombin time (s)		12 (12–15.5)	12 (12–15.5)	12 (12–15)	0.469	
Prothrombin activity (%)		100 (65.9–100)	100 (65.9–100)	100 (65.8–100)	0.469	
International normalized ratio		1 (1–1.36)	1 (1–1.4)	1 (1–1.39)	0.473	
Activated partial thromboplastin time (s)		35 (33–40)	36 (34–38)	36 (34–37)	0.012*	*P1* = *0.327* *P2* = 0.009* *P3* = *0.630*
Thrombin time (s)		19 (18–20)	20 (18–20)	20 (18–20)	0.278	
D-dimer (mg/l)		1.3 (0.6–8)	4.2 (1.2–9.2)	8.1 (0.8–12)	< 0.001*	*P1* = 0.001* *P2* = < 0.001* *P3* = 0.002*

Categorical data are expressed as count (%); parametric data are expressed as mean ± SD; non-parametric data are expressed as median (minimum–maximum). *, * p*< 0.05 is considered significant. P: comparison between 3 groups; ***P1:*** comparison between C1 and C2 groups; P2: comparison between C1 and C3 groups; and P3: comparison between C2 and C3 groups

**Table 3:** Data of CRC cases categorized by tertiles of plasma d-dimer levels

		D-dimer			*p*	Pairwise comparison
		G1 (< 3.5 g/l, n = 35)	G2 (3.6–7.3 g/l, n = 33)	G3 (> 7.4 g/l, n = 32)		
Male		8 (22.9%)	22 (66.7%)	15 (46.9%)	0.003*	*P1* = < 0.001* *P2* = 0.045* *P3* = *0.127*
Female		27 (77.1%)	11 (33.3%)	17 (53.1%)		
Age (years)		60 (36–74)	51 (18–73)	52 (27–77)	0.523	
Site	Colon	22 (62.9%)	20 (60.6%)	20 (62.5%)	0.979	
	Rectum	13 (37.1%)	13 (39.4%)	12 (37.5%)		
Smoking	Positive	7 (20%)	14 (42.4%)	6 (18.8%)	0.051	
	Negative	28 (80%)	19 (57.6%)	26 (81.3%)		
Hypertension	Positive	21 ( (60%)	5 (15.2%)	10 (31.3%)	< 0.001*	*P1* = < 0.001* *P2* = 0.027* *P3* = *0.150*
	Negative	14 (40%)	28 (84.8%)	22 (68.8%)		
Diabetes	Positive	4 (11.4%)	7 (21.2%)	7 (21.9%)	0.454	
	Negative	31 (88.6%)	26 (78.8%)	25 (78.1%)		
Tumor stage	T1	1 (2.9%)	2 (6.1%)	0 (0%)	< 0.001*	*P1* = 0.006* *P2* = < 0.001* *P3* = 0.029*
	T2	6 (17.1%)	2 (6.1%)	0 (0%)		
	T3	28 (80%)	20 (60.6%)	13 (40.6%)		
	T4	0 (0%)	9 (27.3%)	19 (59.4%)		
Node stage	N0	24 (68.6%)	1 (3%)	3 (9.4%)	< 0.001*	*P1* = < 0.001* *P2* = < 0.001* *P3* = 0.001*
	N1	11 (31.4%)	21 (63.6%)	5 (15.6%)		
	N2	0 (0%)	11 (33.3%)	23 (71.9%)		
	N3	0 (0%)	0 (0%)	1 (3.1%)		
Metastasis stage	M0	35 (100%)	29 (87.9%)	22 (68.8%)	0.001*	*P1* = *0.056* *P2* = < 0.001* *P3* = *0.076*
	M1	0 (0%)	4 (12.1%)	10 (31.3%)		
Stage	I	3 (8.6%)	0 (0%)	0 (0%)	< 0.001*	*P1* = < 0.001* *P2* = < 0.001* *P3* = *0.172*
	II	20 (57.1%)	4 (12.1%)	3 (9.4%)		
	III	12 (34.3%)	25 (75.8%)	19 (59.4%)		
	IV	0 (0%)	4 (12.1%)	10 (31.3%)		
Histological Grade	G1	0 (0%)	0 (0%)	2 (6.3%)	0.012*	*P1* = 0.042* *P2* = 0.005* *P3* = *0.052*
	G2	28 (80%)	20 (60.6%)	23 (71.9%)		
	G3	7 (20%)	8 (24.2%)	1 (3.1%)		
	G4	0 (0%)	5 (15.2%)	6 (18.8%)		
CA19-9 (U/ml)		56 (13–214)	213 (112–1800)	776 (213–3400)	< 0.001*	*P1* = < 0.001* *P2* = < 0.001* *P3* = 0.002*
CEA (ng/ml)		65 (13–219)	210 (113–1780)	912 (472–1800)	< 0.001*	*P1* = < 0.001* *P2* = < 0.001* *P3* = 0.004*
Platelet count (× 10^9^/L)		312 (127–937)	286 (53–747)	283 (153–619)	0.271	
Prothrombin time (s)		12 (12–15.5)	12 (12–15.5)	12 (12–15)	0.607	
Prothrombin activity (%)		100 (65.9–100)	100 (65.9–100)	100 (65.8–100)	0.607	
International normalized ratio		1 (1–1.4)	1 (1–1.4)	1 (1–1.36)	0.594	
Activated partial thromboplastin time (s)		35 (33–39)	35 (34–40)	36 (34–38)	0.359	
Thrombin time (s)		20 (18–20)	20 (18–20)	20 (18–20)	0.923	
Fibrinogen (mg/dl)		258 (210–552)	367 (310–532)	487 (267–567)	< 0.001*	*P1* = < 0.001* *P2* = < 0.001* *P3* = 0.003*

Categorical data are expressed as count (%); parametric data are expressed as mean ± SD; non-parametric data are expressed as median (minimum–maximum). *, * p*< 0.05 is considered significant. P: comparison between 3 groups; P1: comparison between G1 and G2 groups; P2: comparison between G1 and G3 groups; and P3: comparison between G2 and G3 groups

Univariate analysis identified fibrinogen (OR = 1.018, *p* < 0.001), d-dimer (OR = 1.140, *p* < 0.001), and activated partial thromboplastin time (OR = 1.205, *p* = 0.031) as predictors of CRC severity. Multivariate regression confirmed d-dimer (OR = 1.102, *p* < 0.001) and fibrinogen (OR = 1.002, *p* < 0.001) as independent predictors of CRC severity (Table 4).

**Table 4:** Univariate and multivariate analyses of clinical and laboratory variables and aggressiveness of CRC

		Regression						
		Univariable			Multivariable			
		*p*	OR	95% CI	*p*		OR	95% CI
Age		0.014*	0.980	0.964–0.996		0.023*	0.995	0.992–0.998
Sex	Males		1	Reference				
	Females	0.067	0.655	0.417–1.031				
Special habits	smoking	0.775	0.930	0.565–1.530				
Comorbidities	DM	0.788	1.081	0.611–1.912				
	Hypertension	0.004*	0.505	0.316–0.806		0.510	0.975	0.953–1.102
Histological grade	G1 + G2		1	Reference				
	G3 + G4	0.272	1.326	0.801–2.194				
Laboratory	CA19-9	< 0.001*	1.027	1.017–1.036		0.392	1.013	0.992–1.034
	CEA	0.310	1.130	0.892–1.432				
	Platelet	0.552	1.000	0.998–1.001				
	INR	0.773	1.387	0.151–12.733				
	APTT	0.031*	1.205	1.018–1.427		0.725	1.006	0.971–1.043
	TT	0.166	1.226	0.919–1.634				
	Fibrinogen	< 0.001*	1.018	1.014–1.022		< 0.001*	1.002	1.001–1.003
	d-dimer	< 0.001*	1.140	1.126–1.153		< 0.001*	1.102	1.078–1.127

OR, odds ratio; CI, confidence interval

## Discussion

VTE in the context of CRC is difficult to manage. Thromboembolic events are a common cause of death amongst patients with CRC, even in patients who have a good cancer prognosis. VTE is a significant predictor of death within 1 year of cancer diagnosis. It is a particularly strong predictor in patients with local or regional disease, suggesting that VTE may be associated with biologically more aggressive cancers [14].

There is a growing body of evidence supporting a symbiotic relationship between the clotting system and the biology of cancer. CRC leads to increased activation of the clotting system, whilst certain coagulation proteins, e.g. tissue factor (TF), have upregulated expression on CRC tumors. It is possible that this leads to biologically more aggressive cancers, leading to poorer outcomes [15].

Emerging evidence suggests that the coagulation system may have a role in the development and progression of cancer [16, 17]. The current study set out to examine the relationship between routine coagulation markers and colorectal cancer (CRC) stages, confirming a strong correlation between CRC invasiveness and the coagulation markers d-dimer and fibrinogen.

Plasma d-dimer and fibrinogen were identified as independent risk factors for advanced-stage CRC which is associated with poorer prognosis and unfavorable clinicopathological characteristics. Incorporating these coagulation markers into the current CRC risk assessment models could help clinicians plan devise more effective treatment plans and suitable follow-up strategies.

Fibrinogen is a 340 kDa soluble glycoprotein composed of three peptide chains linked by 29 disulphide linkages, is crucial in the coagulation cascade. It may facilitate CRC progression through multiple mechanisms. First, it forms a protective fibrin shield around the tumor cells, shielding them from immune responses while providing structural support to the extracellular matrix a robust. This enables the effect of fibroblast growth factor and vascular endothelial growth factor, promoting angiogenesis and tumor growth [18]. Second, fibrinogen receptors on the tumor cells enable adhesion to endothelial cells, aiding vascular invasion and metastasis [19]. Additionally, fibrinogen can enhance tumor cell-platelet aggregation via β3-integrin-mediated pathways, facilitating immune evasion and further dissemination [20].

Previous reports, indicate that absence of fibrinogen significantly reduces the metastatic potential of invasive tumor, particularly those that spread through the blood and lymphatic systems [21]. Cancer cells also release fibrinogen [22] which can be deposited without thrombin cleavage [23]. Fibrin/fibrinogen deposition activates fibrinolysis activity, breaking down extracellular matrix. Elevated plasma fibrinogen levels have been identified as prognostic indicators in various cancers, including esophageal [24], stomach [25], uterus [26] and ovarian cancer [27]. In non-small cell lung or stomach cancer [28], high fibrinogen plasma levels correlate with poor survival and disease severity [29].

In CRC, elevated plasma fibrinogen levels have been associated with worse survival [30] Chen et al. [31]. Tang et al. [32] found that high preoperative fibrinogen level is linked to distant metastases and a poor prognosis. Hepatocytes synthesize fibrinogen, which is transformed into fibrin by activated thrombin Xa [33]. Prior studies have also reported a positive correlation between CRC stage, tumor size, and plasma fibrinogen levels [24, 25, 28, 30, 34]. Furthermore, Xie et al. suggested that fibrinogen could predict high bone metastasis burden in prostate cancer [35].

D-dimer is a marker of fibrinolysis and a byproduct of cross-linked fibrin degradation, rises with increased fibrinolytic activity. Elevated d-dimer levels have been observed in various cancers, including stomach [36], CRC [31, 37], lung [38, 39], ovarian [40] and breast cancer [41]. Oya et al. [42] demonstrated a correlation between d-dimer level and the extent of tumor invasion in CRC. In this study, higher preoperative d-dimer levels were found in advanced TNM stages of CRC.

The mechanism behind the increase in plasma fibrinogen and d-dimer levels in CRC cases with adverse clinicopathological characteristics remains unclear. CRC cells may upregulate fibrinogen production or release cytokines that elevate plasma fibrinogen levels. Additionally, cancer cells may suppress the fibrinolysis resulting in elevation of these markers [43]. Advanced-stage CRC cells may have an enhanced ability to synthesize fibrinogen and cytokines. This hypothesis is yet to be evaluated in future research.

This study found no significant correlation between CRC severity and prothrombin time, prothrombin activity, thrombin time, or international normalized ratio (INR). These findings align with prior research [30, 44]. However, Ferrigno et al. [45] reported that prolonged prothrombin time was associated with a poor prognosis in lung cancer.

Platelet activation is critical for coagulation function. Malignant tumors can disrupt coagulation, leading to thrombocytosis [46, 47]. Platelets protect tumors from immune responses, release growth factors, and promote angiogenesis, aiding tumor progression [48, 49]. Thrombocytosis has been linked to poor prognosis in solid tumors. Reducing platelet counts may hinder tumor growth and metastasis [50]. However, the relationship between platelet count and CRC severity remains debated. While Sasaki et al. [51] found an association between thrombocytosis and tumor progression, our study aligns with findings from Lee et al. [30], which showed no significant correlation.

Variability in inclusion criteria, patient populations, and laboratory methodologies may contribute to discrepancies among studies. While prothrombin time primarily reflects the extrinsic coagulation pathway, activated partial thromboplastin time (APTT) represents the intrinsic and common pathways. INR standardizes prothrombin time across laboratories [52], and thrombin time measures fibrinogen-to-fibrin conversion [53]. Prothrombin time and APTT have been linked to recurrence-free survival in breast cancer [54], and thrombin time correlates with five-year survival in esophageal squamous cell carcinoma [55]. High INR levels have also been associated with poor survival in epithelial ovarian cancer [56]

This study has limitations. First, it was conducted at a single center, limiting generalizability. Second, coagulation parameters were not reassessed after initial evaluation. Additionally, this study did not assess the predictive value of fibrinogen and d-dimer in relation to CRC survival due to a lack of prospective follow-up.

In conclusion, our findings highlight a strong association between CRC aggressiveness and plasma fibrinogen and d-dimer levels, suggesting these biomarkers could aid in assessing CRC progression. While the underlying mechanisms remain unclear, this study provides a foundation for future research.

## Supplementary Information

Additional file1 (DOCX 137 kb)

## Data Availability

All data will be available by the corresponding author in a reasonable request.

## Abbreviations

AJCC: American Joint Committee on Cancer
CA: Carbohydrate antigen
CEA: Carcinoembryonic antigen
CRC: Colorectal cancer
IRB: Institutional review board
MRI: Magnetic resonance imaging
OCMU: Oncology center Mansoura university
TNM: Tumor/Node/Metastasis
WHO: World Health Organization
