# Supplementary material for: Coagulation markers as independent predictors of colorectal cancer aggressiveness
Source: BMC Gastroenterol. 2025 Sep 2;25:634. doi: 10.1186/s12876-025-04249-4 (PMC12403284; doi:10.1186/s12876-025-04249-4)
Supplement: Supplementary file 1 — Additional file1 (DOCX 137 kb) [file 12876_2025_4249_MOESM1_ESM.docx]

**Supplemental Data:**

**Supplemental table 1: Clinicopathological characteristics and coagulation markers among different TNM stages of CRC cases.**

|  | |  | **CRC AJCC/TNM pathological Staging** | | | ***p*** | ***Pairwise comparison*** |
| --- | --- | --- | --- | --- | --- | --- | --- |
|  |  | **Early disease (stages I & II)**  **(N=30)** | | **Node Metastasis (stage III)**  **(N=56)** | **Distant Metastasis (stage IV**  **(N=14)** |  |  |
| **Male** | | 14(46.7) | | 25(44.6%) | 6(42.9%) | 0.932 |  |
| **Female** | | 16(53.3%) | | 31(55.4%) | 8(57.1%) |  |  |
| **Age (years)** | | 61(27-74) | | 54(18-76) | 34(27-77) | 0.095 |  |
| **Site** | **Colon** | 21(70%) | | 31(55.4%) | 10(71.4%) | 0.302 |  |
|  | **Rectum** | 9(30%) | | 25(44.6%) | 0(0%) |  |  |
| **Smoking** | **Positive** | 6(20%) | | 21(37.5%) | 0(0%) | **0.011*** | *P1=0.143*  *P2=0.155*  ***P3=0.007**** |
|  | **Negative** | 24(80%) | | 35(62.5%) | 14(100%) |  |  |
| **Hypertension** | **Positive** | 13(43.3%) | | 23(41.1%) | 0(0%) | **0.010*** | *P1=0.839*  ***P2=0.003****  ***P3=0.003**** |
|  | **Negative** | 17(56.7%) | | 33(58.9%) | 14(100%) |  |  |
| **Diabetes** | **Positive** | 3(10%) | | 15(26.8%) | 0(0%) | **0.026*** | *P1=0.096*  *P2=0.540*  ***P3=0.030**** |
|  | **Negative** | 27(90%) | | 41(73.2%) | 14(100%) |  |  |
| **Histological Grade** | **G1** | 2(6.7%) | | 0(0%) | 0(0%) | **0.013*** | ***P1=0.039****  *P2=0.274*  ***P3=0.018**** |
|  | **G2** | 23(76.7%) | | 34(60.7%) | 14(100%) |  |  |
|  | **G3** | 4(13.3%) | | 12(21.4%) | 0(0%) |  |  |
|  | **G4** | 1(3.3%) | | 10(17.9%) | 0(0%) |  |  |
| **CA19-9 (U/ml)** | | 43(13-1200) | | 214(112-3400) | 741(324-1800) | **<0.001*** | ***P1= <0.001****  ***P2= <0.001****  *P3=0.051* |
| **CEA (ng/ml)** | | 56(13-1700) | | 217(112-1800) | 915(472-1800) | **<0.001*** | ***P1= <0.001****  ***P2= <0.001****  *P3=0.065* |
| **Platelet count (×10^9^/L)** | | 271(164-549) | | 324(53-937) | 234(171-343) | **0.016*** | *P1= 0.107*  *P2= 0.169*  ***P3=0.007**** |
| **Prothrombin time (s)** | | 12(12-15.5 | | 12(12-15.5) | 12(12-12) | 0.184 |  |
| **Prothrombin activity (%)** | | 100(56.9-100) | | 100(65.9-100) | 100(100-100) | 0.184 |  |
| **International normalized ratio** | | 1(1-1.39) | | 1(1-1.4) | 1(1-1) | 0.168 |  |
| **Activated partial thromboplastin time (s)** | | 35(33-39) | | 35(34-40) | 36(35-37) | **0.030*** | *P1= 1.000*  ***P2= 0.025****  *P3=0.094* |
| **Thrombin time (s)** | | 20(18-20) | | 20(18-20) | 19(18-20) | 0.308 |  |
| **Fibrinogen (mg/dl)** | | 246(210-552) | | 388(265-567) | 479(410-532) | **<0.001*** | ***P1= <0.001****  ***P2= <0.001****  ***P3=0.010**** |
| **D-dimer (mg/l)** | | 1.2(0.6-12) | | 4.5(2.2-9.3) | 8.1(6.3-9.3) | **<0.001*** | ***P1= <0.001****  ***P2= <0.001****  ***P3=0.031**** |

Categorical data are expressed as count (%); parametric data are expressed as mean±SD; non-parametric data are expressed as median (minimum-maximum). *, p<0.05 is considered significant. ***P:*** comparison between 3 groups; ***P1:*** comparison between early and node metastasis; ***P2***: comparison between early and distant metastasis groups; and ***P3***: comparison between node and distant metastasis groups.

**Supplemental Figure 1:**

| 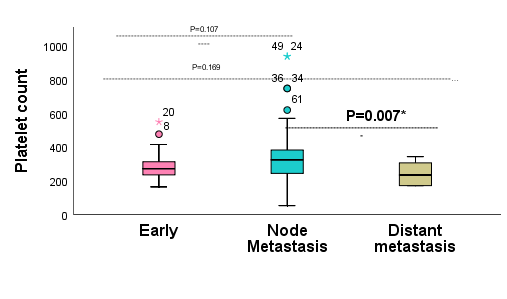 | 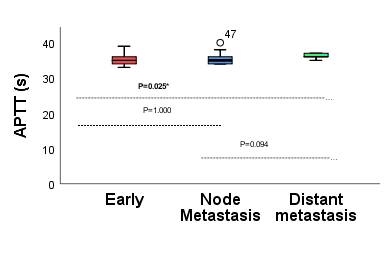 |
| --- | --- |
| 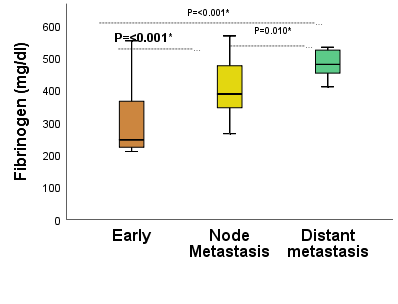 | 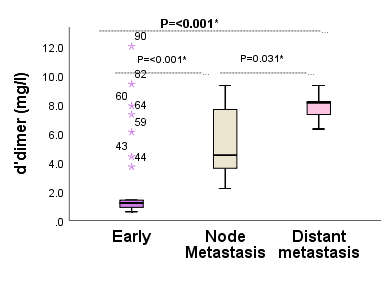 |

**Comparison of coagulation markers among different TNM CRC stages.**

| 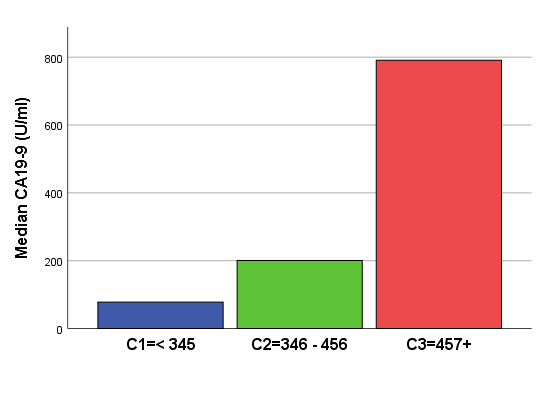 | 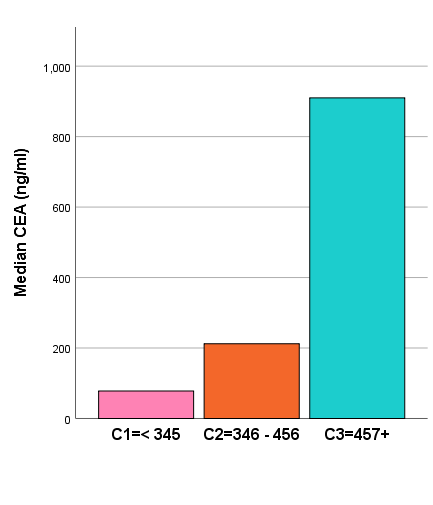 |
| --- | --- |
| 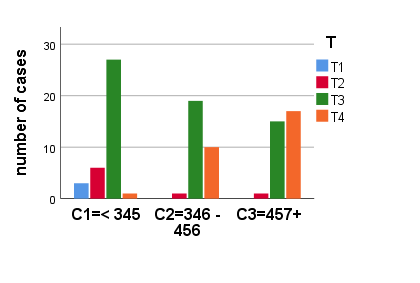 | 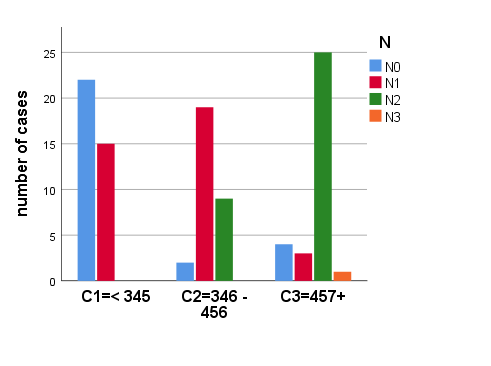 |
| 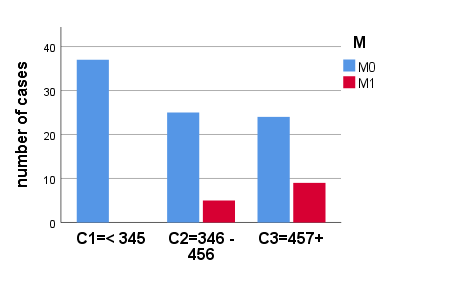 | 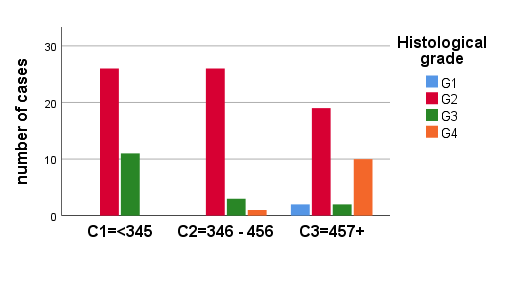 |

**Supplemental Figure 2: Clinicopathological traits in CRC cases stratified into tertiles by plasma fibrinogen levels.**

| 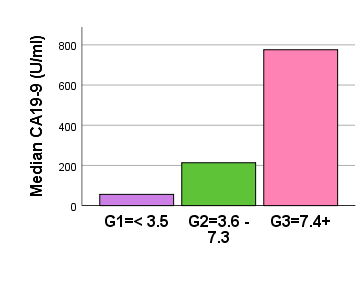 | 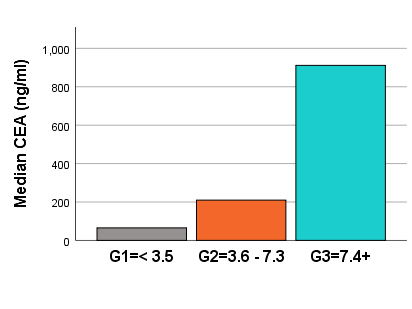 |
| --- | --- |
| 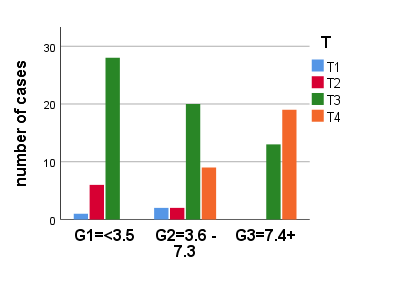 | 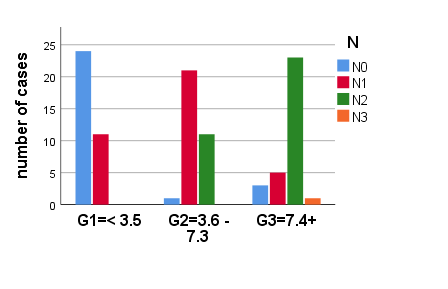 |
| 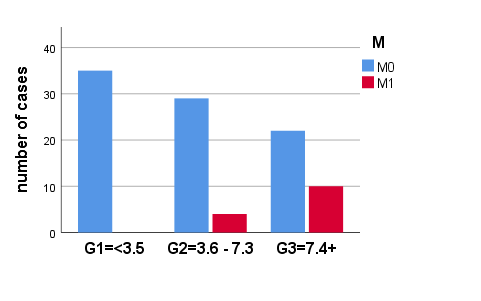 | 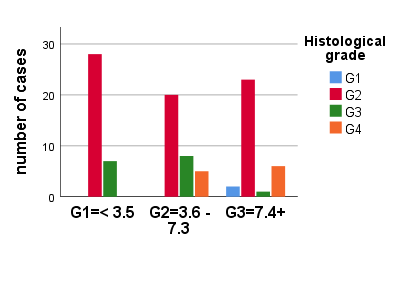 |

**Supplemental Figure 3: Clinicopathological traits in CRC cases stratified into tertiles by plasma d-dimer levels.**

**Supplemental Figure 4: Flow diagram of the study**
